# Incidence and remission of endometriosis in Germany based on prevalence data from 35 million patients from the statutory health insurance

**DOI:** 10.1186/s12905-026-04615-8

**Published:** 2026-06-23

**Authors:** Maryam Schütte Saem, Sabrina Voß, Tobias Pytlik, Sven Schiermeier, Ralph Brinks, Katja Ickstadt

**Affiliations:** 1 Faculty for health (Department for human medicine), University of Witten/Herdecke, Alfred-Herrhausen-Straße 50, 58448 Witten, NRW Germany; 2 Faculty of Statistics, TU Dortmund University, Vogelpothsweg 87, 44227 Dortmund, NRW Germany; 3 Faculty for health, University of Witten/Herdecke, Marienplatz 2, 58452 Witten, NRW Germany

**Keywords:** Endometriosis, Illness-Death Model, Prevalence, Partial differential equation (PDE), Women’s health, Epidemiology

## Abstract

Endometriosis is a chronic gynecological disease that can potentially develop as early as birth and can restrict the life of the patient. With the increasing prevalence of endometriosis in Germany, it has recently gained attention and priority for consideration. While numerous studies have examined endometriosis in Germany and other countries, studies on its incidence and remission rates are rare. Therefore, this study aimed to estimate the incidence and remission rates of endometriosis based on its age-specific prevalence in German women from 2012 to 2022.

This study utilized data from the Central Institute (Zi) for Statutory Health Insurance (SHI) in Germany [1], which observed more than 35 million SHI-insured adolescents and women aged ***≥*** 10 years in 2012 and 2022, to determine the age-specific prevalence of ascertained diagnoses of endometriosis. The Illness-Death Model was used to estimate the incidence and remission rates of endometriosis. The estimation is based on a bootstrapping approach with 5000 replicates-samples. Results are the median incidence and remission rates, reported with 95% confidence intervals (CIs) based on 2.5% and 97.5% percentiles-quantiles, which quantify the uncertainty of the estimated incidence and remission rates.

The bootstrapping approach estimated the incidence (median incidence across all bootstrap samples) about 1.73 per 1,000 person-years at around age of 32 years, and the highest remission rate was about 70.04 at the age of 52 years. The estimated 95% (CI) for the incidence and remission rates were (95% CI 1.71–1.78) and (95% CI 68.1–75.0), respectively.

The Illness-Death Model and the prevalence of endometriosis from the Zi enabled the incidence rate of ascertained endometriosis for German women to be estimated. This study was the first to estimate the remission rate of endometriosis in Germany, aiming to better understand the condition.

**Supplementary Information:**

The online version contains supplementary material available at 10.1186/s12905-026-04615-8.

## Introduction

Endometriosis is one of the most common gynecological diseases among girls and women, globally affecting 5%–10% of women of reproductive age [2–4]. In endometriosis, some tissues similar to those lining the uterus can grow outside the uterus, causing pain during menstruation. Currently, there is no curative treatment for endometriosis. Management focuses on symptom control and includes analgesics, hormonal therapies such as progestins and gonadotropin-releasing hormone (GnRH) agonists or antagonists, and, in some cases, surgery [5]. These treatments aim to reduce disease activity and symptoms, although their effects are reversible after discontinuation. GnRH-based therapies may differ in their pharmacological profiles, potentially affecting recovery of reproductive function after treatment cessation. Infertility in endometriosis is multifactorial and is often related to adhesions and chronic inflammatory processes that can impair reproductive function.

Endometriosis is classified as a chronic disease with the ICD-10-GM code N80 in the International Classification of Diseases, where GM refers to the German modification [6]. Like other chronic conditions, predicting the prognosis for patients with endometriosis can be challenging. However, the Illness-Death Model (IDM) has been used in forecasting the outcomes of chronic diseases such as diabetes [7, 8] and for estimating the long-term care needs of individuals who are dependent on care [9]. Brinks and Landwehr [10] developed a formula based on partial differential equations (PDEs) that describes the relationship between mortality, incidence, and prevalence and can be used to estimate the prevalence and incidence of chronic diseases. One aspect of endometriosis that has not been sufficiently explored is its potential for remission. While it is unlikely that those with chronic diseases will become symptom-free, many patients with endometriosis experience a reduction in their symptoms after menopause. However, there remains a dearth of studies in Germany and worldwide investigating the incidence of endometriosis, with even fewer exploring its remission rate.

A German study by Abbas et al. [11] in 2012 used data from women insured by AOK Hesse, a German health provider, to estimate the prevalence and incidence of endometriosis among women aged 15–54 years. Similarly, a Danish cohort study [12] analyzed women aged 15–55 years diagnosed with endometriosis between 1990 and 2017, using data from the Danish National Patient Registry to estimate the prevalence and incidence of endometriosis in the Danish female population. Christ et al. [13] analyzed data from a diverse cohort of U.S. women diagnosed with endometriosis from January 2006 to December 2015. They focused on women aged *≥* 10 years, encompassing the typical reproductive age range for endometriosis diagnosis, estimating both the prevalence and incidence of endometriosis within this population.

However, a research gap that remains is the lack of consideration of endometriosis as a chronic disease with the potential for remission, which brings hope for symptom improvement in affected patients. Therefore, utilizing PDEs derived from the IDM and incorporating the findings from Kohring et al. [1] on the prevalence of endometriosis among German women, we derived the incidence and remission rates of endometriosis in this population. To our knowledge, this study is the first to estimate the remission rate and one of the few to assess incidence rather than prevalence, which represents an important contribution to understanding the epidemiology of endometriosis.

The remainder of this article is organized as follows. We first describe the methodological framework, including the mathematical and statistical approaches. Next, we present the estimated incidence and remission rates. We then compare our findings with previous studies and discuss the strengths and limitations of the study. Finally, we summarize the main conclusions and provide an outlook for future research. Unless otherwise stated, all incidence and remission rates are reported per 1,000 person-years.

## Methods

### Data

This study used a prevalence dataset from research by Kohring et al. [1], encompassing female patients aged 10 to 70 years in Germany, who were covered under statutory health insurance (SHI) and had a documented diagnosis of endometriosis from 2012 to 2022. The data for this study are from Versorgungsatlas, a research initiative by the Central Institute for Statutory Health Insurance Physicians (Zi) in Germany, which provides detailed analyses and reports on healthcare services and their regional distribution across the country. Endometriosis cases were identified using the ICD-10-GM coding system with the diagnostic code N80.

According to Fig. 2, between 2012 and 2022, a total 35,602,313 females aged 10 years and older with SHI were monitored, out of which 339,718 were diagnosed with endometriosis. The median age of the diagnosed patients included in this study was 40 years. The prevalence proportion of diagnosed endometriosis among females increased from 5.7 to 9.5, representing a 65% relative increase across the decade. The median age of patients diagnosed with endometriosis between 2012 and 2019 was 42 years, with a slight decrease to 40 years in 2022, indicating some variation in prevalence by age group. The diagnosis prevalence increased in 2022, particularly among younger women aged 10–14 years, peaking at 21.9 for women aged 35–39 years. Notably, the diagnosis prevalence increased across all age groups, especially between the ages of 20 and 54 years. The estimated crude prevalence of endometriosis was 16.8 cases. In 2012, the highest prevalence (14.8) was observed among women aged 40–44 years, while by 2022, the age peak shifted, signaling a general increase in diagnoses across all age groups over time. We conclude that the observed prevalence of endometriosis is lower than anticipated, attributing this discrepancy to a significant degree of underdiagnosis.

### Illness-death model

**Fig. 1:**
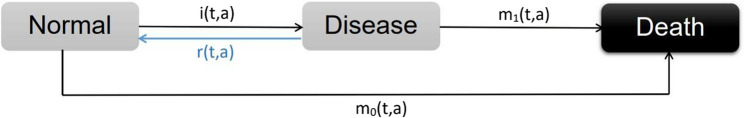
Illness-Death Model with three states and transition rates *i*,* m*_0_, *m*_1_, and *r* depending on calendar time *t* and age *a*

The IDM provides a framework for describing a population according to a disease, using three states: *Healthy*, *Diseased*, and *Dead* (Fig. 1). Subjects in the underlying population are in one of these three states. Key metrics in the IDM are the age-specific prevalence, the incidence rate, the remission rate, and the mortality rate. Age-specific prevalence *p*(*t*,* a*) represents the proportion of diseased people aged *a* at time *t.* Transitions between states are the incidence rate (*i*), which is the rate of new cases, the mortality rate without disease (*m*_0_), the mortality rate with disease (*m*_1_), and the remission rate (*r*) (the reduction of symptoms). These rates depend on calendar time *t* and age *a*.

Brinks and Landwehr [10] established a PDE framework for assessing the prevalence of a chronic disease using the IDM. Their approach aimed to estimate both the incidence and prevalence proportion using Eq. (1):

1 $$\left(\frac{\partial}{\partial t}+\frac{\partial}{\partial a}\right)\;p=\left(1-p\right)\;\left(i-p\triangle m\right)-pr,$$

where *t* and *a* are the variables for calendar-time and age, and ∆*m* = *m*_1_- *m*_0_ (For further details on the partial differential equations (PDE), please see the supplementary file 1, Sect. 1.).

The Danish Atlas of Disease Mortality [14] shows that the ratio of mortality between women with and without endometriosis (mortality rate ratio (MRR)) can be assumed to be 1 (MRR = 0.95, 95% CI: 0.9–1.01), indicating that the excess mortality of endometriosis is close to 0, which means that there is no difference in mortality between these two groups as both have similar/comparable mortality rates. According to Breslow and Day [15], the MRR is stable across populations and, therefore, the MRR can also be applied to Germany, which leads to the conclusion ∆*m* = *m*_1_- *m*_0_ = 0.

Given prevalence data from two cross-sections, we employ a method for estimating the incidence *i* and the remission *r* at the midpoint between the cross-sections in year *t*_0_ by using the following approaches:

2 $$i\left(t_0,a;\beta\right)\;:=i\left(a;\beta\right)=\beta_0\;exp\left(- \left\{ \frac{a-\beta_1}{\beta_2} \right \} ^2\right),$$

3 $$r\left(t_0,a;\gamma\right)\;:=r\left(a;\gamma\right)=\gamma_0\;exp\left(- \left\{ \frac{a-\gamma_1}{\gamma_2} \right \} ^2\right)\cdot$$

We can estimate the coefficients *β* = (*β*_0_, *β*_1_, *β*_2_) for *i* and *γ* = (*γ*_0_, *γ*_1_, *γ*_2_) for *r* by a least-squares estimator. For this, we select ages *a*_*k*_, for *k* = 0,*…*,* n*, from the age range and set (*∂p*)_*k*_ := *∂p*(*t*_0_, *a*_*k*_), *p*_*k*_ := *p*(*t*_0_, *a*_*k*_), *i*_*k*_(*β*) := *i*(*t*_0_, *a*_*k*_; *β*), and *r*_*k*_(*γ*) := *r*(*t*_0_, *a*_*k*_; *γ*). Then, the least-squares estimation minimizes the following functional:

4 $$\sum\limits_{k=0}^n\left[\left(\partial p\right)_k-\left\{\left(1-p_k\right)\times\;i_k\left(\beta\right)-p_k\times\;r_k\left(\gamma\right)\right\}\right]^2\rightarrow\;\min\limits_{\beta,\gamma}\cdot$$

### Statistical methods

In this study, incidence and remission are not directly observed but are inferred from modeled age-specific prevalence using the illness–death model. Estimates are therefore obtained as continuous functions of age rather than as counts of newly diagnosed cases within discrete age groups. The incidence and remission rates of endometriosis among German women are estimated using the prevalence dataset from [1]. In the first step, the prevalence is modeled using a polynomial regression model based on data collected from 2012 to 2022 (see supplementary file 1, Sect.3 for additional information about polynomial regression). This model predicts the prevalence for *t*_0_ = 2017. We selected 2017 as it represents the midpoint between 2012 and 2022, providing a balanced reference for our analysis. By minimizing the sum of squares in Eq. (4), which was derived from the IDM and the related PDE, the coefficients *β* and *γ* of the incidence and remission rates for the year *t*_0_ = 2017 are estimated [16].

To account for uncertainty in the prevalence data, incidence and remission are estimated using a bootstrapping approach where the age-specific incidence and remission rates are calculated in 5,000 bootstrap samples [16]. The median across these 5,000 samples estimates the age-specific incidence and remission. To assess the accuracy and precision of the estimation, a 95% bootstrap CI was calculated as the 2.5th and 97.5th quantiles of the resulting bootstrap samples as the lower and upper bounds, respectively

All analyses, including all figures and calculations, were generated using the statistical software R (version 4.4.2) (R Core Team, 2024).

## Results

### Prevalence estimation

**Fig. 2:**
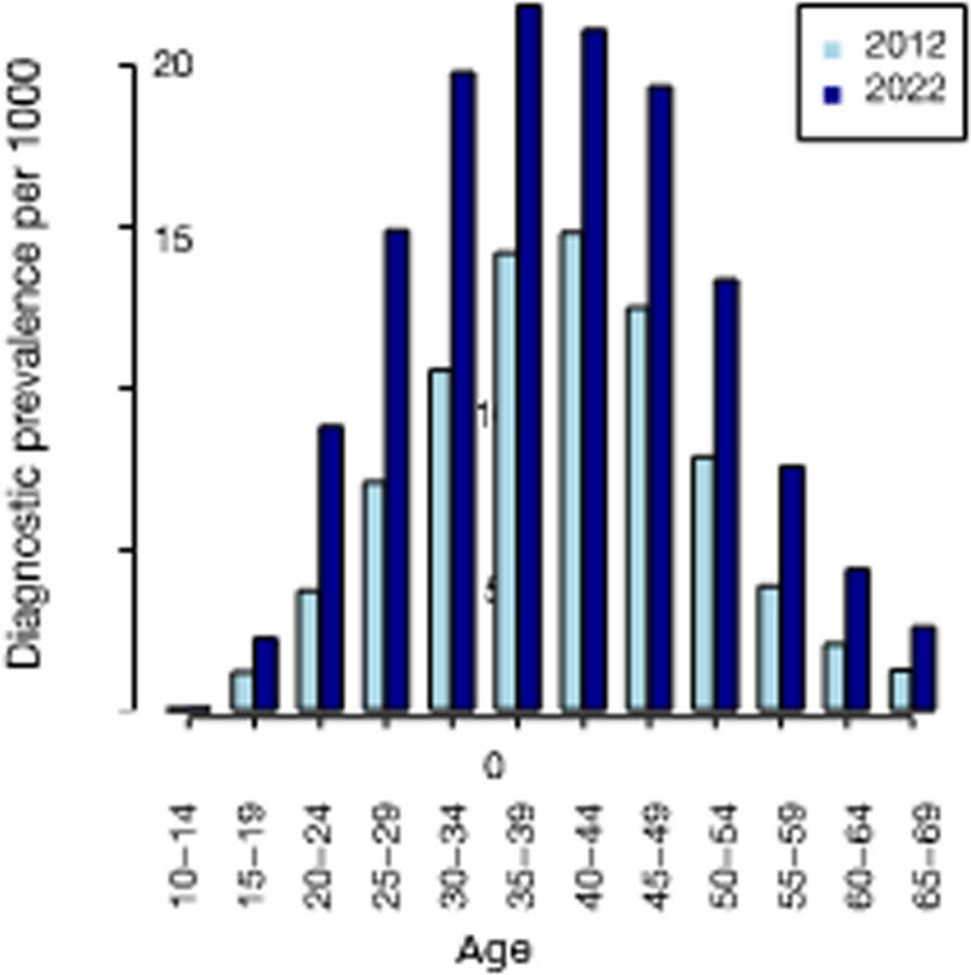
Observed prevalences of endometriosis in Germany from the Central Institute for Statutory Health Care for 2012 and 2022 for 5-year age groups of 5 years

The prevalence data recorded by the Central Institute for Statutory Health Care in Germany include females aged 10–70 years. In 2012, the peak prevalence was below 15 cases, while by 2022, the peak had risen to 22 cases (Fig. 2).

**Fig. 3:**
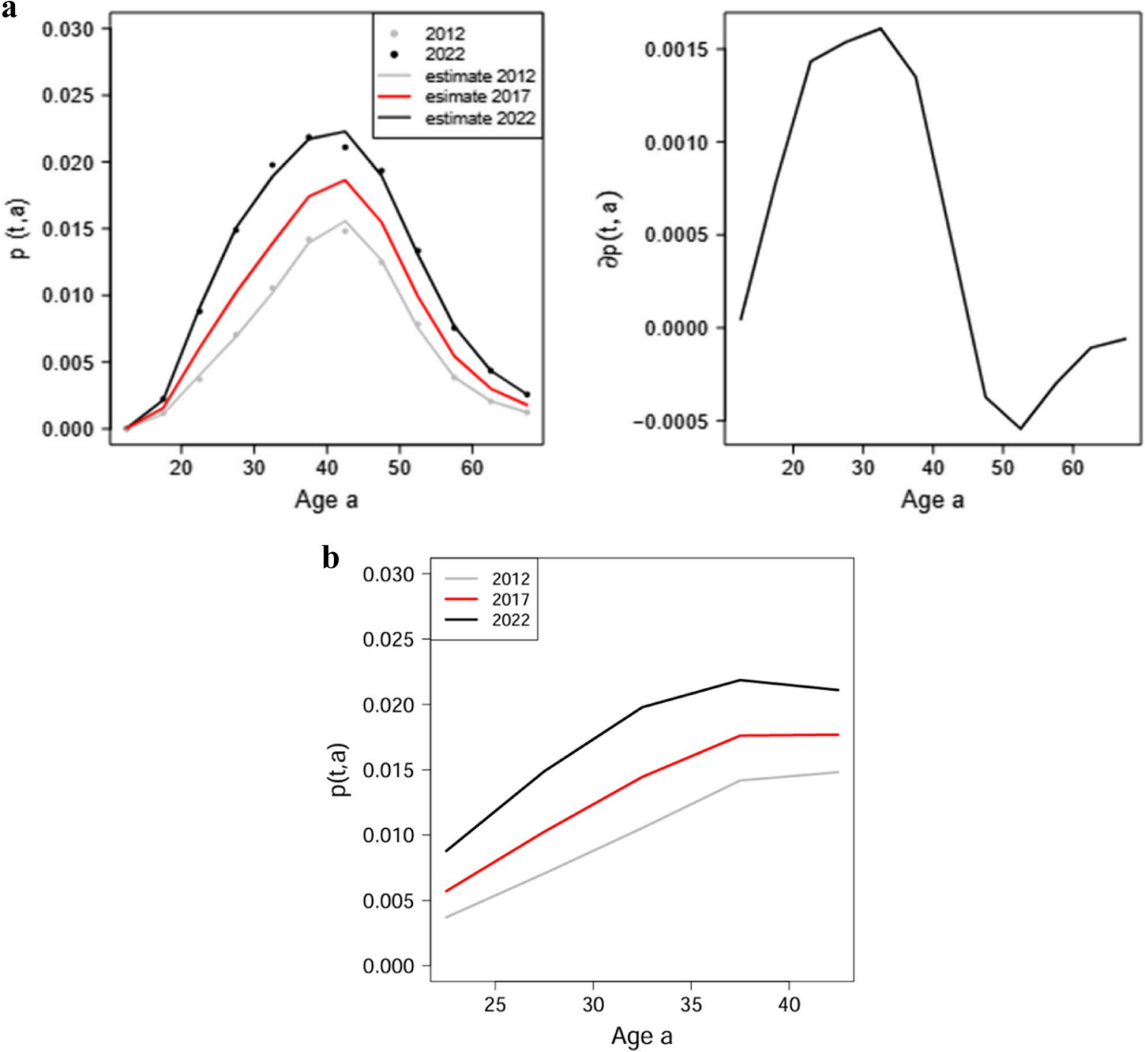
**a** Left: Estimated prevalence values p(t, a), for the year 2017 by interpolation of the data for 2012 and 2022. Right: The age-specific partial derivative *∂p* for year *t* = 2017. **b** Estimated prevalence values p(t, a), for the year 2017 by interpolation of the data for 2012 and 2022 for the age group 20–44

These prevalence data are modeled using a polynomial regression to calculate prevalences for other years. Figure 3a (left) shows the overall population age-specific prevalence estimated with this model for 2012 (gray), 2017 (red), and 2022 (black) as lines. For 2012 and 2022, the observed prevalences are shown as gray and black points, respectively. A visual comparison demonstrates that the model accurately reflects the observed prevalences, reinforcing its suitability.

The prevalence for 2017 initially rises steadily, reaching a value of 1% by the age of 28 years, indicating that 1% of women of this age had the diagnoses with endometriosis in that year. The diagnosis prevalence continues to increase, peaking at approximately 1.8% around the age of 42 years. After this peak, the prevalence begins to decrease, so that by the age of around 52 years, only 1% of women have endometriosis condition. The connection between the peak prevalence observed in this study and clinical practice can be largely attributed to the fact that young women are frequently advised that dysmenorrhea is a normal occurrence and does not require further investigation.

According to the IDM and Eq. (1), the derivative of prevalence is analyzed as well, as the incidence and remission rates are incorporated into this calculation. The right side of Fig. 3a shows that the prevalence growth reaches its highest point around the age of 32 years, with an increase of about 1.6. As noted earlier, the rate of increase becomes negative between the ages of 42 and 47 years. The changes in the derivative of prevalence in (1) can be explained in relation to both incidence and remission rates. 

Up to the age of 45 years, the derivative of prevalence is positive, as the incidence follows the Gaussian function (2) that produces these positive values. Therefore, this shows that the incidence is increasing, leading to more cases of endometriosis. After the age of 45 years, the derivative is negative due to the Gaussian function (3) describing the remission rate, which dominates after this age. This leads to fewer cases of endometriosis.

The greatest decrease in prevalence, around 0.5, is observed at approximately 52 years of age. After this age, the decline in prevalence slows down but does not completely plateau.

### Incidence and remission rate estimation

**Fig. 4:**
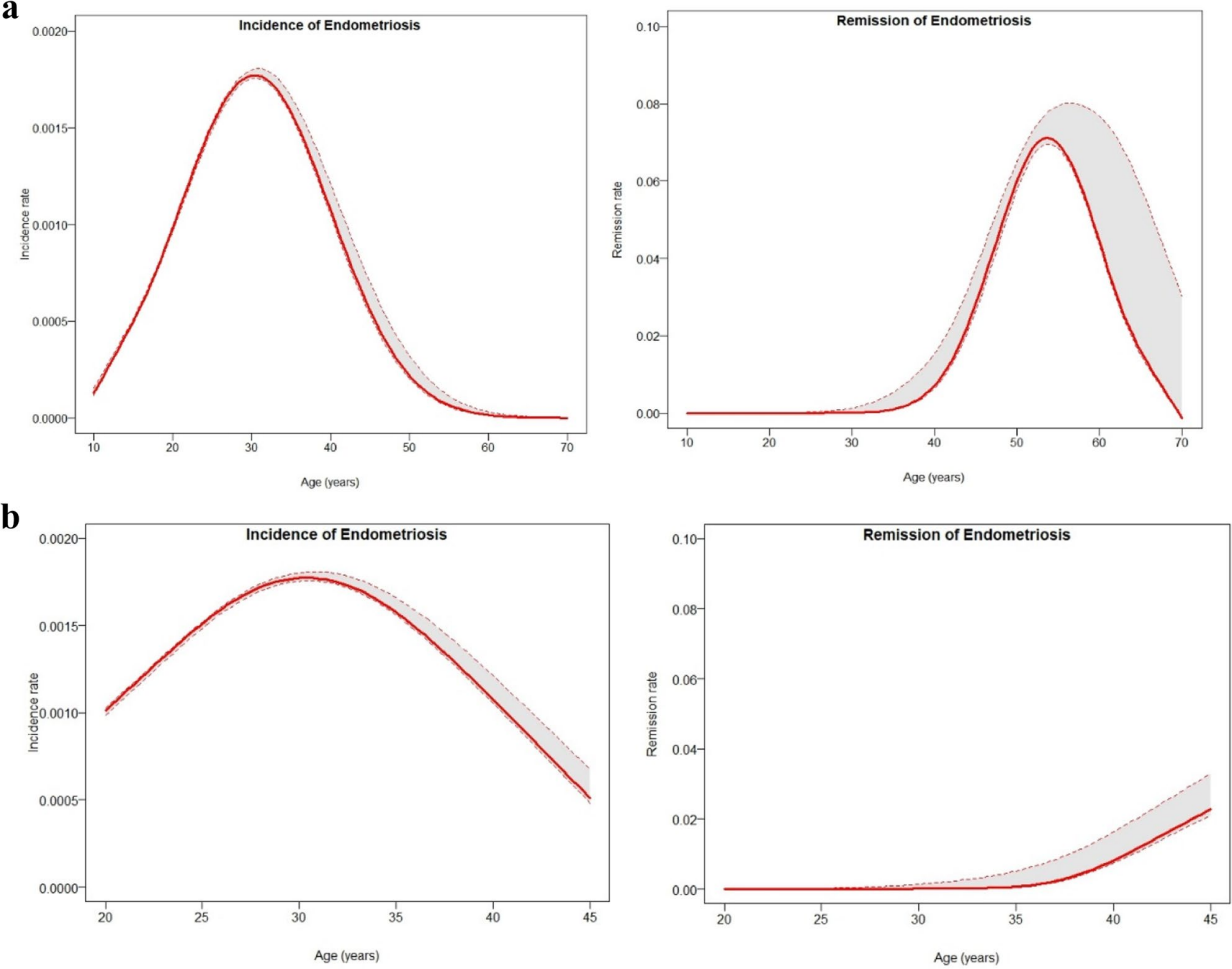
**a** Left: Estimated median incidence rates from 5000 bootstraps with pointwise 95% CIs. Right: Estimated median remission rates using the same approach **b** Left: Estimated median incidence rates for the age group 20–44 from 5000 bootstraps with pointwise 95% CIs. Right: Estimated median remission rates for the age group 20–44 using the same approach

The incidence and remission rates can be estimated from the derivative of prevalence, Eq. (1). To assess the accuracy of the estimated incidence and remission rates, we applied a bootstrapping approach to the prevalence data from 2012 to 2022 (see Sect. Statistical methods). Figure 4a shows the median incidence in the 5,000 bootstrap samples with a 95% CI as the estimated incidence. The incidence increases steadily from the age of 13 years, reaching approximately 0.07% by the age of 18 years. The estimated incidence peaks around age 30–32 years, reaching a maximum of 1.73. As shown in Fig. 4a, the incidence rate increases steadily until the age of 27 years. The median incidence peaks between the ages of 27 and 32 years at 1.695 to 1.730, respectively. After the age of 32 years, the median incidence starts to decline, reaching approximately zero by around the age of 57 years, with a median incidence of 0.032.

The analysis of remission indicates that by the age of 47 years, women tend to experience fewer endometriosis symptoms, with a remission rate of 44.48. The highest estimated remission rate of 70.04 occurs around the age of 52 years. After this age, symptoms generally decline, although they may not disappear entirely. Figure 4a illustrates the remission rate of endometriosis, showing that remission starts around the age of 42 years, increasing steadily and peaking around the age of 52 years at 70.04 (95% CI: 68.17-75.00). After this peak, the remission rate declines, with the median dropping to approximately 27.8 by the age of 62 years. After this age, the remission rate estimates become less reliable due to limitations in data quality for older age groups.

#### Prevalence, incidence and remission rate in subgroup of women aged 20–44

In 2017, prevalence among women of reproductive age (20–44 years) steadily increased with age, starting at 0.6% around age 20, exceeding 1% in the late twenties, and reaching a peak of 1.8% by age 43. The age-related pattern was similar in 2012 and 2022, with prevalence increasing across the same age range (Fig. 3b).

Incidence rates are highest within this age group. Median incidence rises rapidly from early adulthood, starting around 0.1%, peaks between ages 27 and 32 (0.16%–0.17%), and remains elevated throughout the third and fourth decades. In contrast, remission rates stay low through much of this period (0%–0.2% for ages 22–38) and increase to 1.5% by age 42, indicating that disease dynamics in women aged 20–44 are primarily driven by incidence rather than remission (Fig. 4b).

## Discussion

### Reflecting on the findings given existing knowledge

Delayed diagnosis of endometriosis is common, as many women initially manage symptoms such as menstrual pain without seeking medical evaluation. Clinical diagnosis often occurs later in the disease course, which may partly explain increasing prevalence estimates over time. Improved awareness through patient organizations and specialized centers may also contribute to higher diagnosis rates.

Our study is one of the few to estimate the age-specific incidence and the first to estimate remission rates of endometriosis in Germany based on prevalence data using the IDM and a corresponding PDE. To validate our estimates, we applied a bootstrapping method with 5,000 repetitions. The estimated incidence rate (median of 5,000 bootstrap samples) was 1.73 (95% CI: 1.71–1.78) around the age of 32 years, demonstrating a narrow CI, which confirms the reliability of our estimates. The median remission rate was 70.04, with a wider CI (95% CI: 68.1–75.0), likely due to data limitations affecting the precision of the remission rate estimation.

Several studies have estimated the prevalence and incidence of endometriosis using different methodologies and populations. Utilizing the IDM, our study is notable in both methodology and the number of individuals in the underlying dataset. Abbas et al. [11] analyzed 62,323 German women with SHI in 2007, reporting an age-standardized prevalence of 0.81% (peaking at 1.28% at age 35–44 years) and an incidence of 3.5 (peaking at 5.0). Based on a smaller dataset, their study used Wilson’s score intervals, a method less suited for large datasets like ours, which may limit its robustness. Christ et al. [13] examined 332,056 women from Kaiser Permanente Washington (2006–2015), identifying 2,863 new cases of endometriosis, with incidence rates declining from 3.12 in 2006 to 1.74 in 2015 among women aged 36–45 years. The prevalence was 1.9% in 2015. Our median incidence of 1.73 is closer to the lower end of the incidence rates reported by Christ et al., indicating that our estimated incidence rate is consistent with their findings but with greater precision due to the larger sample size and the use of advanced statistical techniques like bootstrapping for validation. Rowlands et al. [17] tracked 13,508 Australian women from 1996 to 2018. By the age of 44 years, around 11% had a clinical or suspected diagnosis of endometriosis, with most diagnoses occurring in their early thirties. The incidence increased from 0.2 at the age of 20–24 years to 6 at the age of 30–34 years. They reported a higher incidence, particularly among women in their thirties, than our study. This difference may reflect differences in case definition, data sources, and diagnostic practices between the studies rather than sample size alone.

In 2022, Illum et al. conducted a cohort study in Denmark with 2,188,720 women using data from 1990 to 2017 [12]. They identified 26,605 cases of endometriosis, reporting an incidence rate of 0.789 (95% CI: 0.780–0.799) and a prevalence of 1.63% in 2017. The highest incidence was observed between the ages of 25 and 35 years, peaking at 1.995. Unlike our study, which used the IDM, they employed Cox regression to estimate disease incidence. Cox regression models the hazard of a first diagnosis over time, whereas the IDM models transition between healthy, diseased, and death states simultaneously. This framework allows incidence, prevalence, and other disease parameters to be estimated consistently within a single model.

Medina et al. [18] examined 2.4 million women in Catalonia, Spain (2006–2018), identifying 16,258 cases of endometriosis. By 2018, the overall prevalence had increased to 0.0124, and the median incidence rate was 0.949, peaking in women aged 35–44 years. Our estimated incidence in Germany is higher than the median incidence rate reported by Medina et al. in Catalonia, suggesting that the prevalence of endometriosis may vary across regions. Utilizing the IDM, our approach also offers a more robust framework for understanding both incidence and remission rates within the German population. While Medina et al. used logistic and Poisson regressions for their estimations, our study benefits from a larger dataset and using the IDM, which provides more precise and reliable estimates. Therefore, our estimations are likely more accurate because IDM tracks patients over time by modeling transitions between different disease states, whereas logistic regression only estimates the probability of an event at a single point in time without accounting for disease progression [10].

Our findings align with previous studies showing that endometriosis incidence peaks between the ages of 30 and 44 years, although some studies, such as those in Australia, report an earlier peak at 25–35 years. Our estimated incidence rate of 1.73 falls between the lowest rate reported in Denmark (0.789) and the highest rate reported in Australia (6.0), reflecting different diagnostic practices. The strength of our study lies in its use of a larger dataset and the innovative IDM approach, which provides more accurate estimates. IDM is particularly well-suited for studying chronic diseases, as it captures disease progression by modeling transitions between health states and can handle time-to-event data within a multi-state framework, unlike conventional Cox or Poisson regression approaches that typically focus on single events [19].

Our study also uniquely estimates remission rates, an aspect challenging to capture for chronic diseases such as endometriosis. Unlike traditional methods that focus on incidence, such as Cox and Poisson regression, the IDM and the related PDE allow for a comprehensive view of both incidence and remission, offering a clearer understanding of the disease’s long-term course.

### Strengths and limitations

Our study had some strengths and limitations. One strength was its large sample size, which allows for more precise and reliable estimates of measures such as incidence and remission rates. A larger sample size enhances the representativeness of the study population and reduces random error, thereby improving the precision of estimated incidence and remission rates.

Another strength was its use of a statistical method designed to accurately capture real-world dynamics. It utilized the IDM, a powerful tool for analyzing chronic diseases [19, 20]. By incorporating its mathematical framework, specifically PDEs, the IDM enabled us to establish precise relationships between prevalence, incidence, and remission rates for endometriosis. One notable advantage of this approach is its ability to predict disease paths and estimate key disease parameters. Therefore, the IDM is particularly valuable for projecting future trends and understanding the components of chronic disease progression. The other key advantage of the IDM over other methods, such as cohort or longitudinal studies, is its cost-effectiveness [8]. Unlike these methods, which can be expensive and resource-intensive, the IDM offers a more affordable alternative as it can be performed based on cross-sections. The IDM is also less time-consuming, as it does not require long-term follow-up, allowing for efficient estimation of trends and future outcomes within large populations, making it a valuable tool for studying chronic diseases.

Besides these strengths, our study and the method it used to estimate the incidence and remission rates based on the IDM and a related PDE had some limitations that could impact the estimations. Firstly, incomplete or inaccurate data can undermine the reliability of the IDM, such as missing information on patient demographics (age, sex, socioeconomic status); disease status (diagnosis, severity, remission periods); transitions between illness, remission, and relapse; or healthcare utilization (medical visits, treatments, comorbidities). Such issues can lead to biased or invalid estimates of disease incidence, progression, and remission, ultimately affecting the model’s accuracy in chronic disease analysis. Secondly, estimating certain parameters, such as remission rate or specific mortality risks, can be difficult in this method, particularly when these events are rare or poorly documented in the available datasets. Thirdly, the incidence and remission were inferred from cross-sectional prevalence data. Differences in prevalence across age groups may partly reflect cohort effects (e.g., changes in environmental exposures or diagnostic practices across generations), which could influence the estimated transition rates. Moreover, datasets often only include information on patients who have utilized services from SHI-affiliated physicians and received a confirmed diagnosis through this system; those covered by private health insurance are not included. This exclusion may lead to gaps in data coverage. Given the diagnostic challenges discussed, it is reasonable to infer that the prevalence of endometriosis is considerably underreported. Fourthly, the remission rate peaking towards the end of the estimated age range might be explained by the delay in confirming recovery after menopause. Altogether, these limitations contribute to potential inaccuracies in estimating the remission rate, undermining the reliability of our results.

## Conclusion and outlook

This study analyzed data about 339,718 German women aged 10 years and older with statutory health insurance diagnosed with endometriosis between 2012 and 2022 to estimate age-specific incidence and, for the first time, the remission rate of endometriosis. Using a large population-based dataset and the illness–death model with a related PDE framework, our approach provides robust and reliable estimates of disease dynamics in the German population.

Our findings highlight the continued challenge of delayed diagnosis of endometriosis, which contributes to prolonged symptoms and adverse outcomes such as infertility. The diagnostic delay of approximately 7–10 years between symptom onset and diagnosis ultimately creates a cycle of persistent pain that is difficult to interrupt [21]. Earlier recognition through increased awareness and targeted physician training could reduce diagnostic delays and improve timely access to appropriate surgical or conservative treatment. In addition, the substantial financial burden associated with long-term treatment and monitoring underscores the need to address economic as well as medical aspects of care. The observed prevalence suggests that endometriosis may still be underdiagnosed, reinforcing the importance of improving early detection, particularly among younger women.

By providing precise epidemiological estimates of incidence and remission, this study offers valuable information for healthcare professionals and policymakers to develop more effective management strategies and allocate resources more efficiently. Increased awareness of these disease dynamics may also empower women to better understand and manage their condition.

Future research should examine the influence of lifestyle factors, medical interventions, and genetic or epigenetic mechanisms on the incidence and remission of endometriosis. Expanding analyses to include data from privately insured populations could further enhance the accuracy and generalizability of these estimates.

## Supplementary Information

Supplementary Material 1.

## Data Availability

The data are available in a public, open access repository. Data and source code for use with the open-source statistical software R (including data and analysis) for estimation of the incidence of endometriosis in Germany are freely available on Zenodo (see reference and link below). Prevalence input is based on a large German claims dataset and is uploaded as aggregated data that are publicly available and can be downloaded free of charge from the Care Atlas (Versorgungsatlas) website.Data and source code (Zenodo): Mohammadi Saem, M., Voß, S., Pytlik, T., Ickstadt, K., & Brinks, R. (2025). Incidence and remission of endometriosis in Germany based on preva- lence data from 35 million patients from the statutory health insurance. [10.5281/zenodo.14716555] Versorgungsatlas: [https://www.versorgungsatlas.de/fileadmin/ziva\_docs/129/VA-24-01-Endometriose_Finale_Version.pdf]
