# Supplementary material for: Incidence and remission of endometriosis in Germany based on prevalence data from 35 million patients from the statutory health insurance
Source: BMC Womens Health. 2026 Jun 23;26:318. doi: 10.1186/s12905-026-04615-8 (PMC13289242; doi:10.1186/s12905-026-04615-8)
Supplement: Supplementary file 1 — Supplementary Material 1. [file 12905_2026_4615_MOESM1_ESM.docx]

# Partial differential equations

Let us assume that $S(t, a)$ denotes the absolute number of subjects with age $a$ at time $t$ in the health state and $C(t, a)$ the number of cases with age $a$ at time $t$.

We also consider that the disease is contracted only after birth and the functions $S$ and $C$ are sufficiently smooth.


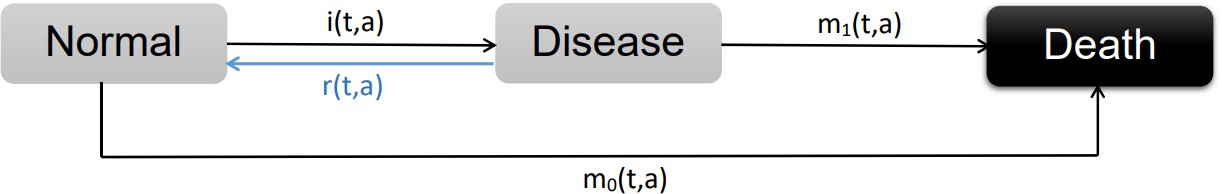


Figure 1: Illness-death model with three states and transition rates *i, m*_0_*, m*_1_ and *r* depending on calendar time *t* and age *a*.

From the IDM, the following balance equations for the functions $S$ and $C$ will be obtained:

$$\left( \partial_{t}+\partial_{a} \right)S\left( t, a \right)= -\left( m_{0}\left( t, a \right)+ i\left( t, a \right) \right)S\left( t, a \right)+ r\left( t, a \right)C\left( t, a \right),$$

$$\left( \partial_{t}+\partial_{a} \right)C\left( t, a \right)= -\left( m_{1}\left( t, a \right)+ r\left( t, a \right) \right)C\left( t, a \right)+ i\left( t, a \right)S\left( t, a \right).$$

The prevalence can be calculated through the following formula:

$p\left( t,a \right)=\frac{C(t,a)}{S\left( t,a \right)+C(t,a)} .$ (1)

By applying derivative on both sides of the above formula, we obtain the PDE

$\left( \partial_{t}+\partial_{a} \right)p=\left( i-\left( m_{1}-m_{0} \right)p \right)\left( 1-p \right)-rp.$ (2)

For the proof, we apply the formula [(1).](#_bookmark0) Therefore, by assuming $\left( \partial_{t}+\partial_{a} \right)=\partial$, we have

$$\partial p=\partial\left( \frac{C}{S+C} \right)=\frac{\partial C\left( S+C \right)-\left( \partial S+\partial C \right)C}{\left( S+C \right)^{2}}=\frac{\left( \partial C \right)S-\left( \partial S \right)C}{\left( S+C \right)^{2}}$$

$$=\frac{-m_{1}SC-rSC+{iS}^{2}+m_{0}CS+iCS-rC^{2}}{\left( S+C \right)^{2}}$$

$$=\frac{iS^{2}-rC^{2}+(m_{0}+i-r)SC}{{(S+C)}^{2}}$$

$=\frac{i\left( S+C \right)^{2}\left( 1-p \right)^{2}-rp^{2}\left( S+C \right)^{2}+\left( m_{0}+i-m_{1}-r \right)p(1-p)\left( S+C \right)^{2}}{\left( S+C \right)^{2}}$

$$=i\left( 1-p \right)^{2}-rp^{2}+\left( m_{0}+i-m_{1}-r \right)p\left( 1-p \right).$$

Now we rearrange the terms

$$\partial p=i-2ip+ip^{2}-rp^{2}+m_{0}p+ip-m_{1}p-rp-m_{0}p^{2}-ip^{2}+m_{1}p^{2}+rp^{2}$$

$=i\left( 1-p \right)-\left( m_{1}-m_{0} \right)p+{\left( m_{1}-m_{0} \right)p}^{2}-rp$

$$=i\left( 1-p \right)-\left( m_{1}-m_{0} \right)p\left( 1-p \right)-rp$$

$$=\left( i-\left( m_{1}-m_{0} \right)p \right)\left( 1-p \right)-rp.$$

If $\Delta m:= m_{1}-m_{0}$, then

$\left( \partial_{t}+\partial_{a} \right)p=\left( i-p\Delta m \right)\left( 1-p \right)-rp.$ (3)

By knowing the incidence rate $i$, mortality rates $m_{0}$, $m_{1}$, remission rate $r$ and by applying the above PDE, the prevalence $p$ can be calculated at time $t$ and age $a$.

We assume that the mortality rates without disease $m_{0}$ and with disease $m_{1}$ are not known and the remission rate is zero. Then, using the general mortality m of the overall population with $m=pm_{1}+(1-p)m_{0}$ and the relative mortality $R=\frac{m_{1}}{m_{0}}$, Equation [(3)](#_bookmark1) reads

$\left( \partial_{t}+\partial_{a} \right)p=\left( 1-p \right)\left( i-m\frac{p(R-1)}{p\left( R-1 \right)+1} \right).$ (4)

The advantages of PDE [(4)](#_bookmark2) become evident when information on $m_{0}$ and $m_{1}$ is not available, and instead general mortality $m$ and relative mortality $R$ are provided; the calculation can be seamlessly performed to determine prevalence $p$ using the associated PDE formula.

It can be realized that the PDE formula is sometimes more practicable to estimate the age-specific incidence rate $i$, because equation [(3)](#_bookmark1) can be directly solved for the incidence (assuming $r$ = 0). Given the prevalence $p$ (with its derivative) and mortality rates $m_{1}$ and $m_{0}$, we obtain

$$i=\frac{\left( \partial_{t}+\partial_{a} \right)p}{1-p}+p\left( m_{1}-m_{0} \right).$$

This formula is useful to approximate the incidence based on data from two cross-sectional studies.

# Mathematical approach

## Prevalence derivative and estimating the incidence and remission rates

For estimating the incidence $i$ and remission rate $r$, we can use the partial differential equation

$$\left( \partial_{t}+\partial_{a} \right)p=\left( i-p\Delta m \right)\left( 1-p \right)-rp,$$

where $t$ and $a$ are calendar time and age variables, and $\Delta m$ is the difference between the mortality with disease and the mortality without disease. By assuming that $\Delta m=r=0$, we get

$$i=\frac{\left( \partial_{t}+\partial_{a} \right)p}{1-p}.$$

In contrast, if we assume that $\Delta m=i=0$, then we have the following formula for the remission rate

$$r=-\frac{\left( \partial_{t}+\partial_{a} \right)p}{p}.$$

These two formulas are used in the mathematical approach of our research.

# Statistical approach

## Polynomial regression model

Polynomial regression extends linear regression to model relationships where the effect of the independent variable $x$ on the dependent variable $y$ is non-linear. This is achieved by including terms of higher degrees (e.g., $x^{2},x^{3}$). The general polynomial regression model is expressed as:

$$y_{i}=\beta_{0}+\sum_{j=1}^{k} \beta_{j}x_{i}^{j}+\epsilon_{i}, i=1,2,\ldots,n,$$

where $y_{i}$ is the dependent variable, $k$ is the degree of the polynomial, $x_{i}$ is the predictor variable, $\beta_{j}$ is the regression coefficient to be estimated and $\epsilon_{i}$ is the random error term, which is assumed to be independent and normally distributed.

To ensure computational stability, polynomial terms are constructed to be orthogonal using the condition:

$$\int_{a}^{b} P_{n}\left( x \right)P_{m}\left( x \right)dx=0,$$

for $m\neq n$. It means, $<P_{n}\left( x \right),P_{m}\left( x \right)> =0$. Orthogonality ensures that adding higher-degree terms does not affect the estimates of lower-degree terms, improving numerical stability and interpret ability.

The polynomial regression model can be extended to include additional predictors and interaction terms, like

$$y_{i}=\beta_{0}+\sum_{j=1}^{k} \beta_{1,j}x_{1,i}^{j}+\sum_{l=1}^{k} \beta_{2,l}z_{i}^{l}+\beta_{3}x_{2,i}+\epsilon_{i} .$$

Here $z_{i}$ is an additional predictor like ”years” which is treated linearly if it has only two levels and the interaction terms $\beta_{3}x_{2,i}$ allow the combined effects of variables to be modeled.

## Matrix formulation

For simplicity, the model can be expressed in matrix form

$$Y=X\beta+\epsilon,$$

where $Y$ is the vector of dependent variables, $X$ is the design matrix with columns representing the predictors and their polynomial terms, $\beta$, vector of regression coefficients and $\epsilon$, the vector of random errors.

Regression coefficients are estimated using the least squares method, minimizing the sum of squared residuals

$$\epsilon^{T}\epsilon=\left( Y-X\beta\right)^{T}\left( Y-X\beta\right).$$

Differentiating with respect to $\beta$ and setting the derivative to zero yields the normal equation

$$X^{T}X\beta=X^{T}Y ,$$

and the solution for $\beta$ is given by

$$\beta={(X^{T}X)}^{-1}X^{T}Y .$$

In summary, this framework is particularly useful for modeling non-linear relationships, ensuring stability through orthogonal polynomial terms, and enabling accurate predictions via the least squares estimator.
